# Magnetic resonance imaging features of Familial Mediterranean Fever associated spondyloarthritis

**DOI:** 10.1186/1546-0096-13-S1-P114

**Published:** 2015-09-28

**Authors:** A Turan, R Mercan, B Bitik, H Kucuk, MA Ozturk, A Tufan

**Affiliations:** 1 Yildirim Beyazit T&R Hospital, Radiology, Ankara, Turkey; 2 Gazi University, Rheumatology, Ankara, Turkey

## Background

Inflammatory back pain is a frequent complaint among Familial Mediterranean Fever (FMF) patients and spondyloarthritis is a well-known chronic manifestation of disease affecting about 10% of patients. However, literature is lacking for a systematic study investigating radiologic features of this particular patient group.

## Objectives

To determine Magnetic Resonance Imaging (MRI) characteristics of patients with FMF associated spondyloarthritis (FMF-SPA).

## Methods

Twenty-nine patients followed up in our clinic with FMF-SPA who fulfilled ASAS classification criteria for axial spondyloarthritis. To figure out only characteristics of FMF-SPA, we excluded those patients with psoriasis, Crohn disease/ulcerative colitis or positive HLA-B27 tests. Patient demographics, clinical features and MEFV mutation analyzes were recorded. All patients underwent sacroiliac and spinal contrast enhanced MR examination. T1, T2 weighted images (WI), STIR sequence and post-contrast fat saturated T1 WI were used to define MRI features.

## Results

The mean (min-max) age of patients was 32.8±7.7 (19-53) years and 55.2% were female. Age at the onset of the inflammatory back pain was 20.5±5.5 (10-30) years and age at the diagnosis was 29±5.4 (22-35). The duration of symptoms was 11.9±8.6 (1-28) years at the time of MR examination. M694V mutation was the most commonly observed MEFV mutation in FMF-SPA patients (75.9%). In sacroiliac joint, active lesions were evident in 19 patients and sacroiliac joint involvement was bilateral in 20 of them. Spinal lesions were quite rare. The most common finding in the axial skeleton was facet joint arthritis.

## Conclusions

Our results confirmed the role of M694V on risk of spondyloarthritis development in patients with FMF. Unlike ankylosing spondylitis, spinal chronic lesions were quite uncommon, even those patients with substantial disease duration and severe sacroiliac joint involvement. Therefore, these results suggest that FMF-SPA might be a distinct type in spondyloarthropathy spectrum of diseases.

